# Boosting Oxygen Evolution Reaction Catalyzed by Transition Metal Carbides

**DOI:** 10.3390/nano15171319

**Published:** 2025-08-28

**Authors:** Xun Zhang, Aiyi Dong, Haiyang Gao, Guanyingze Wang, Yan Yin, Li Che, Honglin Gao

**Affiliations:** 1 Transportation Engineering College, Dalian Maritime University, Dalian 116026, China; 2 School of Science, Dalian Maritime University, Dalian 116026, China; 3 Marine Engineering College, Dalian Maritime University, Dalian 116026, China

**Keywords:** transition metal carbides, electrocatalyst, water splitting, oxygen evolution reaction

## Abstract

In the water splitting process for sustainable hydrogen production, the oxygen evolution reaction (OER) stands as one of the pivotal half-reactions. Nevertheless, the sluggish four-electron transfer process inherent to OER has emerged as a kinetic bottleneck that impedes water electrolysis. To address this challenge, researchers have been devoting substantial efforts to developing high-performance OER electrocatalysts. Currently, iridium (Ir)-based or ruthenium (Ru)-based oxides are widely acknowledged as benchmark catalysts for OER. However, their scarcity and exorbitant cost render large-scale applications impractical. In recent years, transition metal carbides have garnered extensive attention in the realm of OER electrocatalysts, exhibiting tremendous application prospects owing to their advantages of low cost, high catalytic activity, and excellent stability. This review briefly introduces the fundamental characteristics and synthesis methodologies of transition metal carbides, summarizes the recent research advances in their application as OER catalysts, elaborates on the modification strategies and catalytic mechanisms of transition metal carbide nanomaterials, and finally discusses the challenges confronted by these metal carbides as well as the future research directions.

## 1. Introduction

Over the past century, the extensive exploitation and utilization of fossil energy have strongly driven the unprecedented development of human civilization. However, their excessive consumption is triggering a dual crisis: environmental pollution (such as the intensification of global warming caused by greenhouse gas emissions) and energy depletion (the sharp reduction in reserves of non-renewable resources) [1,2,3]. To build a sustainable energy system, developing new energy storage and conversion technologies to replace traditional fossil fuels has become a global consensus [4,5,6,7]. Among them, hydrogen is widely recognized as the most promising energy carrier due to its ultra-high energy density and green preparation pathways. Among various hydrogen production technologies, electrocatalytic water splitting stands out for its advantages of pollution-free emissions, no by-products, and high-purity hydrogen production, with its fundamental reaction being 2H_2_O→2H_2_ + O_2_ [8]. This technology can not only realize the conversion and storage of electricity from renewable energy, but also provide core support for the hydrogen energy economy, thus boasting broad application prospects [9,10,11,12].

The hydrogen production process via water electrolysis involves two key half-reactions: the hydrogen evolution reaction (HER) at the cathode and the oxygen evolution reaction (OER) at the anode [13]. Its theoretical thermodynamic equilibrium voltage is 1.23 V, but due to the limitations of reaction kinetics, an additional overpotential (η) must be applied during actual operation to overcome electrode polarization losses. It is worth noting that compared with the HER process involving two-electron transfer, the OER involves four consecutive proton-electron coupling steps and needs to overcome a higher Gibbs free energy barrier (usually >1.70 eV) [14,15]. This results in its reaction kinetics being significantly slower than that of HER, thus making it the dominant factor in the entire reaction. Therefore, the slow kinetics of OER have become the decisive factor restricting the overall energy conversion efficiency. To improve the efficiency of hydrogen energy production, there is an urgent need to develop high-performance electrocatalysts at low overpotentials [16]. Currently, noble metal oxides such as IrO_2_ and RuO_2_ are regarded as benchmark materials for OER performance [17,18]. However, their scarce reserves, high cost, and structural deactivation issues under industrial current densities (such as Ir dissolution and Ru oxidation) have severely hindered large-scale commercial applications. Against this backdrop, research focus has shifted to the development of transition metal-based electrocatalysts with abundant resources and excellent cost-effectiveness, such as metal oxides [19], hydroxides [20], phosphides [21,22], chalcogenides [23], nitrides [24], and other metal-free materials.

Transition metals have emerged as ideal candidates for OER catalysts due to their unique electronic structures and physicochemical properties. The unfilled 3d orbitals of these metals can flexibly accept or release electrons, forming variable valence states, thereby efficiently adsorbing and activating OER intermediates and significantly reducing the energy barrier of the four-electron transfer process [25]. Compared with noble metals, transition metals have the advantages of abundant resources and low cost. Moreover, their performance can be further optimized through bimetallic synergy strategies. The designability of their crystal structures allows for the exposure of more active sites and the promotion of mass transfer through strategies such as heterogeneous interface engineering and porous morphology regulation.

In recent years, transition metal carbides (TMCs) have attracted attention due to their unique physicochemical properties, such as high stability, high electrical conductivity, and high mechanical strength. Li et al. [26] synthesized a ternary metal composite NiFeMo_2_C@C, which exhibited high OER catalytic activity and stability in 0.1 M KOH solution. The ternary material NiFeMo_2_C@C only required an overpotential of 292 mV to reach a current density of 10 mA cm^−2^ in 0.5 M KOH electrolyte [26]. Abdullah Al Mahmud and his team synthesized a bimetallic carbide/nitrogen-doped graphene composite catalyst (MoC/NiC@N-doped rGO). Its innovation lies in embedding molybdenum carbide (MoC) and nickel carbide (NiC) into a nitrogen-doped reduced graphene oxide (N-rGO) support. This catalyst exhibits efficient bifunctional catalytic activity, requiring only an overpotential of 298 mV to reach a current density of 10 mA cm^−2^ in 0.5 M H_2_SO_4_, with a Tafel slope of 80 mV/dec [27].

TMCs have attracted considerable attention in the field of energy catalysis due to their unique physicochemical properties. TMCs are interstitial compounds, where carbon atoms occupy the interstitial sites of the transition metal lattice [28]. Their crystal structures are mainly classified into face-centered cubic (FCC), hexagonal close-packed (HCP), and simple hexagonal (SH) structures [29]. The chemical bonds of TMCs consist of a mixture of covalent bonds, ionic bonds, and metallic bonds, which endow them with high electrical conductivity, mechanical stability, and corrosion resistance. The hybridization between the sp orbitals of carbon atoms and the d orbitals of transition metals leads to the broadening of the d-band, resulting in a platinum-like electronic structure.

The traditional high-temperature synthesis methods for TMCs typically involve reacting carbon sources with metals or metal oxides under a protective atmosphere. Although these processes are well-established, they are plagued by issues such as low specific surface area, particle agglomeration, and surface carbon film coverage caused by high temperatures, problems that significantly limit catalytic activity. To optimize material properties, several categories of universal synthesis strategies have been developed so far: The Temperature-Programmed Carbothermal Synthesis (TPCS) enables the reaction between transition metal oxides and flowing hydrocarbon gases through programmed temperature elevation. By regulating the type of carbon source, gas flow rate, and temperature-ramping protocol, the crystal morphology and pore structure can be precisely controlled. However, the hydrocarbon decomposition process in this method tends to introduce polymeric carbon impurities that are difficult to remove. The carbothermal hydrogen reduction method uses carbon supports with high specific surface area (e.g., graphene) to load metal salt precursors, which then undergo direct carbonization in a reducing atmosphere. By leveraging the confinement effect of the carbon matrix, this method suppresses particle aggregation and improves dispersibility. Although it can reduce gaseous carbon contamination, it still has the issue of trace carbon residues. The solid-state reaction method involves the high-temperature reaction of solid-phase carbon sources (such as biomass) with metal compounds. Through in situ carbonization, porous composite structures are constructed, and the pore network of these structures can enhance mass transfer efficiency and expose more active sites. The organic-inorganic hybrid method utilizes metal precursors and organic ligands to form complexes, which then undergo low-temperature pyrolysis to achieve carbonization. This method enables the regulation of material composition through molecular design and allows the acquisition of high-purity products at relatively low temperatures [30].

Based on the current research status in the field, this review first briefly introduces the OER catalytic mechanisms, including the AEM and the LOM. Subsequently, focusing on cobalt carbides, nickel carbides, and iron carbides, and incorporating other multi-component TMCs, it systematically summarizes the latest research progress of these materials in OER. Emphasis is placed on presenting the performance differences of various TMC materials in alkaline, acidic, and neutral electrolytes, as well as the basic physicochemical properties of TMCs and mainstream synthesis methods (such as thermal reduction carbonization, metal-organic framework (MOF)-derived method, electrospinning-thermal treatment method, etc.). Furthermore, it clarifies the action mechanisms of modification strategies for electronic structure regulation—including d-band center shift, heterogeneous interface engineering, and conductive support optimization-in reducing reaction energy barriers and increasing the exposure rate of active sites. Finally, the core challenges faced by TMC-based OER catalysts are sorted out, and potential future research directions are proposed.

## 2. Mechanism

**Figure 1:**
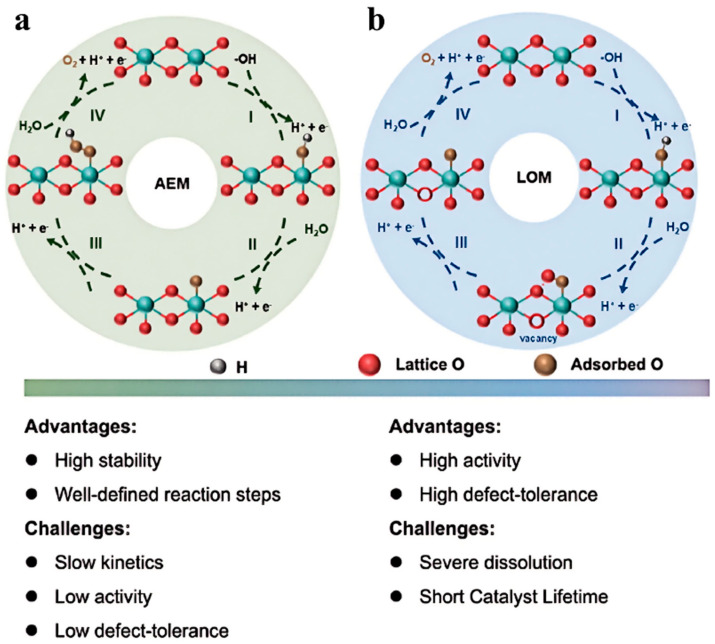
Schematic of potential OER pathways according to (**a**) AEM, (**b**) LOM, and their proposed advantages and disadvantages. Reproduced with permission [31].

Two reaction mechanisms for the OER have been proposed: the adsorbate evolution mechanism (AEM) and the lattice oxygen oxidation mechanism (LOM) (Figure 1).

The AEM suggests that the OER involves a series of concerted electron-proton transfer steps between the catalytically active sites on the electrocatalyst and oxygen intermediates (including OH*, O*, OOH*, and O_2_). There exists a linear scaling relationship in the adsorption energies of these oxygen intermediates, which limits the improvement of the catalytic activity of electrocatalysts [32].

The LOM posits that the oxygen evolution reaction proceeds through the direct generation of OH*, O^2−^, and O_2_. This reaction pathway has kinetic advantages because it bypasses the formation of OOH*, thereby potentially avoiding the high overpotential required in the AEM. However, LOM necessitates the presence of defects such as oxygen vacancies in the electrocatalyst, which can severely damage the crystal structure of the catalyst, leading to extensive dissolution of active sites and consequent instability in reaction performance.

Understanding the mechanism of the OER is crucial for the rational design of efficient OER electrocatalysts.

### 2.1. AEM


(1) $$M^{*}+H_{2}O\to M^{*}\mathrm{OH}+H^{+}+e^{-\;*}$$

(2) $$M^{*}\mathrm{OH}\to M^{*}O+H^{+}+e^{-}$$

(3) $$M^{*}O+H_{2}O\to M^{*}\mathrm{OOH}+H^{+}+e^{-}$$

(4) $$M^{*}\mathrm{OOH}\to M^{*}+O_{2}+H^{+}+e^{-}$$

In the OER, the traditional AEM follows a four-step consecutive surface reaction pathways (1)–(4). It starts with the adsorption of hydroxide ions or water molecules on the active sites (M*) of the catalyst to form OH intermediates, which then undergo deprotonation to generate O intermediates. Subsequently, the O intermediates combine with OH-/H_2_O in the electrolyte to form *OOH intermediates, and finally, oxygen is released through desorption (*OH → O → OOH → O_2_) [33]. The kinetic characteristics of this mechanism can be quantified using density functional theory (DFT) combined with the computational hydrogen electrode method: by analyzing the energies and vibration frequencies of reactants and products, the Gibbs free energy change (ΔG_i_) for each step is calculated, and the step corresponding to the maximum ΔG_i_ is defined as the potential-determining step (PDS). Studies have shown that for most electrocatalysts, step (3) (i.e., the conversion of O → OOH) often becomes the PDS and dominates the theoretical lower limit of overpotential (370 mV). This is because it involves the formation of O-O bonds with high energy barriers and is limited by the scaling relationship of intermediate adsorption energies (ΔG_OOH_ = ΔG_OH_ + 3.2 eV). As the basic theoretical framework for OER, the four-step electron-proton transfer pathway of AEM provides a core model for understanding catalyst activity and guiding catalyst design.

### 2.2. LOM

In the OER, the traditional AEM proceeds through a redox process mediated by metal active sites, involving stepwise electron transfer pathways of intermediates such as OH, O, and OOH. This pathway is constrained by the linear scaling relationship ΔG_OOH_ = ΔG_OH_+ 3.2 ± 0.2 eV, resulting in a theoretical lower limit of overpotential of 370 ± 100 mV. However, the performance of many catalysts has exceeded this limit, indicating the existence of alternative mechanisms, such as the LOM [34,35]. The core difference between AEM and LOM lies in the formation mode of the oxygen-oxygen (O-O) bond: In AEM, the O-O bond is formed by the combination of the O intermediate and OH^−^ in the electrolyte to generate OOH. This process requires electrons to transfer from the adsorbed OH^−^ to the external circuit, and at the same time, electrons from O are injected into metal orbitals. Moreover, the protonation of OOH depends on the electronic properties of the metal state near the Fermi level, creating a kinetic bottleneck. In contrast, LOM forms the O-O bond through the direct coupling of lattice oxygen (O lattice), bypassing the step of OOH generation. Its reaction pathway involves the synergistic effect between oxygen in OH^−^/H_2_O and lattice oxygen. In LOM, after the proton transfer step releases a proton, electrons are directly transferred from the oxygen non-bonding orbitals (O-NB) of O^2−^ to the external circuit, making oxygen the redox center. The electronic states near the Fermi level exhibit the characteristics of oxygen’s non-bonding orbitals [36]. Two conditions must be satisfied simultaneously to activate the LOM: introducing O-NB as buffering orbitals to maintain the initial bond order of metal oxides during electron migration; forming strong metal-oxygen covalent bonds to ensure that electrons are removed from O-NB rather than from the lower Hubbard band, thereby avoiding structural instability [37]. Although the LOM significantly enhances reaction kinetics by circumventing the rate-limiting step of AEM, the consumption of lattice oxygen may lead to the accumulation of oxygen vacancies, triggering structural reconstruction or dissolution of the catalyst and resulting in a loss of stability.

## 3. Cobalt Carbides for Electrocatalytic OER

**Figure 2:**
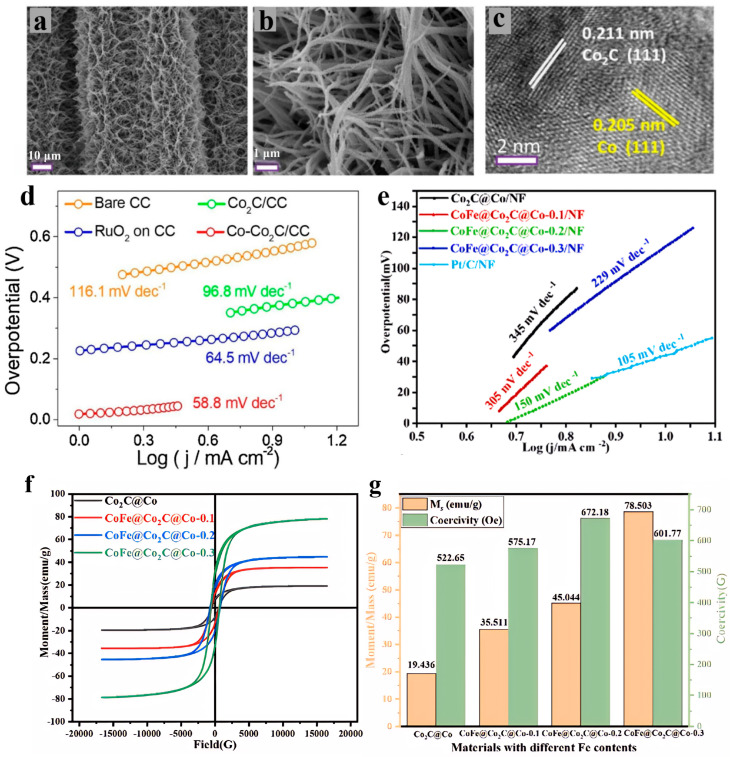
(**a**,**b**) FESEM images of Co-Co_2_C/CC with different magnifications. (**c**) TEM images of Co-Co_2_C material. (**d**) Tafel slopes for different samples. Reproduced with permission [38]. (**e**) The corresponding Tafel curves. (**f**) Hysteresis loops of different samples at room temperature; (**g**) Comparison of saturation magnetization and coercivity of different samples. Reproduced with permission [39].

Cobalt carbide-based materials have emerged as ideal candidates for advancing electrochemical applications across multiple domains, owing to their exceptional mechanical stability, excellent electrical conductivity, high natural abundance, and platinum-like electronic structure (with a d-band center position close to that of noble metals). As efficient bifunctional electrocatalysts, they exhibit comparable activity to noble metals in both the OER and HER. Wang et al. [38] synthesized a heterostructure of Co nanoparticles anchored on Co_2_C nanowires supported by carbon cloth (Co-Co_2_C/CC), as shown in Figure 2a–c. This material demonstrated excellent OER activity in 1 M KOH, requiring an overpotential of only 261 mV at a current density of 10 mA/cm^2^ and exhibiting a Tafel slope as low as 58.8 mV/dec (Figure 2d) [38]. Yang et al. [39] prepared a CoFe alloy/Co_2_C core–shell heterostructure supported on nickel foam (CoFe@Co_2_C@Co/NF). It exhibits excellent bifunctional activity: for OER, it requires an overpotential of 246 mV at a current density of 10 mA/cm^2^ with a Tafel slope of 100 mV/dec; for HER, it needs an overpotential of 53 mV at a current density of 10 mA/cm^2^ with a Tafel slope of 150 mV/dec (Figure 2e–g) [39]. Ritz et al. [40] successfully synthesized Fe_x_Co_1−x_Cy nanocarbides with precisely regulated Fe proportions (x = 0–100%) through the pyrolysis method using Prussian blue analog (PBA) precursors. This material exhibits significant Fe content-dependent OER activity in 1.0 M KOH. The sample with 15–20% Fe shows the lowest overpotential of 420 mV at a current density of 10 mA/cm^2^, with a Tafel slope of 79 mV/dec [40]. As a non-noble metal catalyst for the OER, cobalt carbide, although demonstrating the potential to replace noble metals in alkaline environments, still has core drawbacks such as insufficient intrinsic activity, ineffectiveness in acidic environments, and poor structural stability. To address these issues, recent studies have significantly improved their comprehensive performance through strategies including electronic structure regulation, interface engineering, and carrier optimization.

**Figure 3:**
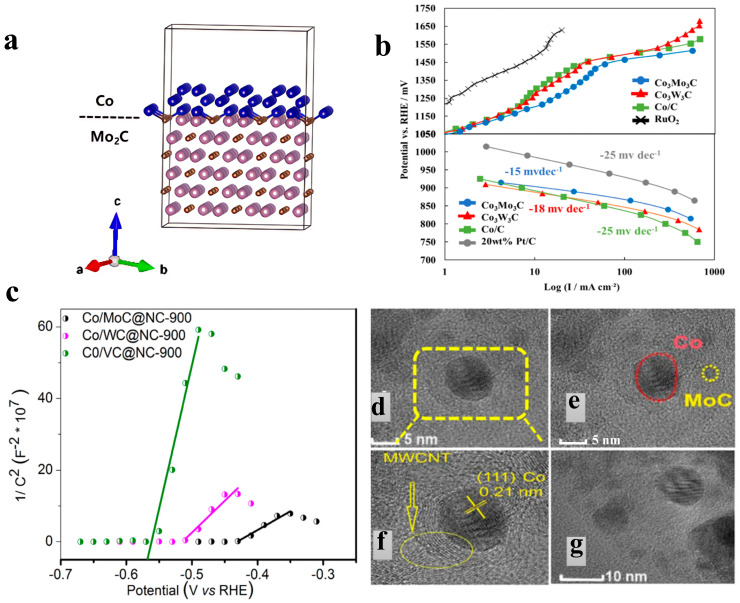
(**a**) Slab geometry of Co (1 1 1)/Mo_2_C (1 0 1) heterointerface for DFT calculation [41]. (**b**) Tafel plots of GDE loaded with Co_3_W_3_C, Co_3_Mo_3_C, and Co/C in 5 mol·L^−1^ KOH at 70 °C. Reproduced with permission [42]. (**c**) Mott–Schottky plots for Co/MoC@NC-900, Co/WC@NC-900, and Co/VC@NC-900 at a fixed frequency of 1000 Hz in 1 M KOH. (**d**–**g**) HRTEM images showing the lattice fringes of Co, MoC, and MWCNT layers. Reproduced with permission [43].

For example, Ahn et al. [41] proposed a strategy using molybdate as a molecular binder to assemble two-dimensional cobalt-based metal-organic frameworks (2D Co-MOFs) into a hydrangea-like three-dimensional structure. After pyrolysis at 900 °C, a composite material was obtained where cobalt/molybdenum carbide heterojunctions are embedded in a nitrogen-doped carbon matrix (H-2D Co/Mo_2_C@NC). This material exhibits excellent OER performance in 1 M KOH, with an overpotential of only 256 mV at a current density of 10 mA/cm^2^, a Tafel slope as low as 48 mV/dec, and stability for 20 h. The core reason for its performance advantages is the electronic structure reconstruction induced by interface engineering. DFT calculations and XPS confirmed (Figure 3a) that the high electronegativity of Mo_2_C drives electron transfer from Co to Mo_2_C, leading to an upward shift of the d-band center of Co and a significant increase in the proportion of high-valence Co^3+^. This electronic reconstruction enhances the adsorption capacity of Co for OH^−^ and reduces the energy barrier of the *O→*OOH step (the rate-determining step), lowering the Tafel slope to 48 mV/dec. In addition, the hydrangea-like morphology provides open active sites, with an electrochemically active surface area of 16.2 mF/cm^2^, and the nitrogen-doped carbon matrix accelerates charge transport, synergistically improving reaction kinetics [41]. Cobalt carbide materials regulate the electronic structure of transition metals through the electron acceptor effect at the interface, driving charge transfer from transition metals to themselves, shifting the metal d-band center upward to optimize the adsorption energy of oxygen-containing intermediates and stabilize the interface. Combined with open morphologies such as hydrangea-like 2D architectures and conductive supports like nitrogen-doped carbon, Co_2_C-based heterojunctions synergistically enhance the exposure of active sites, charge transport, and stability, enabling efficient catalysis with an OER overpotential of <260 mV at a current density of 10 mA/cm^2^. Yanagimoto et al. [42] proposed a strategy for regulating the interface of composite metal carbides. They synthesized Co_3_W_3_C and Co_3_Mo_3_C bimetallic carbides via a wet chemical method. Co_3_Mo_3_C exhibits excellent OER activity in 5 M KOH electrolyte, with an overpotential of 270 mV at a current density of 400 mA/cm^2^, stability exceeding 100 h, and a Tafel slope of 50.6 mV/dec (Figure 3b) [42].

In addition, heteroatom doping in carbon skeletons has been proven to be an effective strategy, as it can optimize the adsorption and desorption processes of intermediates and products while promoting electron transfer. To address the inherent conductivity defects of metal carbide semiconductors, an emerging strategy is to couple them with metal components to construct heterojunctions, thereby significantly enhancing conductivity. To this end, Raj et al. [43] proposed an interface engineering scheme based on the synergy between bimetallic carbides and nitrogen-doped carbon frameworks, and prepared Co/MoC bimetallic carbides supported by graphene/carbon nanotube hybrid carriers (Co/MoC@NC). For this material, the OER overpotential in 1 M KOH is 279 mV at 10 mA/cm^2^, with a Tafel slope of 83.6 mV/dec; in 0.5 M H_2_SO_4_, the OER overpotential is 260 mV at 10 mA/cm^2^, and the Tafel slope is 85 mV/dec. In this material, the nitrogen-doped carbon matrix optimizes the electronic structure of the carbon skeleton by introducing pyridinic nitrogen and pyrrolic nitrogen defects. Specifically, pyridinic nitrogen reduces the work function of the carbon matrix, increases the electron density at the Fermi level, enhances the conductivity of the electrode, and creates active sites for HER/OER. Meanwhile, the polarity induced by nitrogen doping improves hydrophilicity, facilitating mass transfer at the electrode-electrolyte interface. The double Mott–Schottky junctions realize dynamic charge regulation through the interfacial electron redistribution between Co/NC and MoC/NC. Firstly, the MoC/NC junction: The n-type NC injects electrons into MoC. Mott–Schottky tests show that the flat-band potential of Co/MoC@NC shifts positively to −0.43 V (Figure 3c), which increases the d-band electron density of MoC and optimizes the H adsorption energy. Secondly, Co/NC junction: Metallic Co transfers electrons to NC to form a space charge layer, synergistically enhancing interfacial charge transfer. XPS results show that the binding energy of Co 2p shifts positively by 0.8 eV, while that of Mo 3d shifts negatively by 0.5 eV. Thirdly, Co/MoC synergy: As shown in Figure 3d–g, the close contact between Co nanoparticles and MoC promotes electron flow from Co to MoC, further optimizing the adsorption of reaction intermediates [43].

Guo et al. [44] proposed a support optimization strategy by coupling dual-active Co-CoO heterojunctions (metallic Co for promoting HER and CoO for promoting OER with highly conductive and stable two-dimensional Ti_3_C_2_-MXene, synthesizing Co-CoO/Ti_3_C_2_-MXene. This material exhibits excellent bifunctional performance in 1 M KOH. For HER, it shows an onset potential of only 8 mV, overpotentials of 45 mV@10 mA/cm^2^ and 203 mV@100 mA/cm^2^, and a Tafel slope of 47 mV/dec. For OER, the overpotentials are 271 mV@10 mA/cm^2^ and 409 mV@100 mA/cm^2^, with a Tafel slope of 47 mV/dec [44].

Wang et al. [45] adopted a stainless steel mesh (SSM) substrate self-supporting strategy and synthesized the catalyst CoNiC/CoNibc/SSM via a two-step method. Specifically, they grew cobalt-nickel basic carbonate precursors on SSM through a hydrothermal process, and then partially decomposed the precursors into CoNi(OH)_x_ and CO_2_ at high temperature. CO_2_, serving as a carbon source, reacted with CoNi(OH)_x_ to in situ generate Co_2_C-NiC nanosheets (CoNiC), thus forming a hierarchical structure (CoNiC/CoNibc/SSM). As a trifunctional catalyst (for HER/OER/ORR), this material exhibited excellent performance in 1.0 M KOH. For HER, it had an overpotential of 117.6 mV@10 mA/cm^2^ and a Tafel slope of 192.5 mV/dec. For OER, the overpotential was 253.4 mV@10 mA/cm^2^ with a Tafel slope of 36.0 mV/dec. For the oxygen reduction reaction (ORR), it showed a limiting current density of 4.51 mA/cm^2^, a half-wave potential of 0.77 V, and a Tafel slope of 111.3 mV/dec. The excellent catalytic performance of CoNiC/CoNibc/SSM stems from its unique structural design and the synergistic effect between components. The CoNibc precursor grown in situ on the SSM substrate undergoes dynamic reconstruction during high-temperature carbonization: the precursor is partially decomposed into CoNi(OH)_x_ and releases CO_2_, which, as a carbon source, reacts with CoNi(OH)_x_ to in situ generate CoC-NiC composite nanosheets (CoNiC) on the surface of CoNibc. XPS analysis revealed mixed valence states of Co^2+^/Co^3+^ and Ni^2+^/Ni^3+^, as well as a characteristic peak of the C-M bond (282.7 eV), indicating significant charge redistribution at the Co_2_C-NiC interface: electrons are transferred from Ni to Co. During the catalytic process, the bimetallic components exhibit clear division of labor and synergy. For HER, Co sites tend to adsorb H intermediates, while Ni sites promote H_2_ desorption, synergistically reducing the reaction energy barrier. For OER, the Co^3+^/Co^2+^ and Ni^3+^/Ni^2+^ redox couples accelerate O-O bond formation through rapid valence cycling. For ORR, Co_2_C promotes the generation of OOH intermediates, and NiC accelerates O-O bond cleavage; the two synergistically increase the limiting current density and reduce the Tafel slope. In addition, the in situ formed oxygen vacancies provide additional active sites, further enhancing the kinetics of oxygen-related reactions [45,46].

Rajan et al. [47] synthesized a bimetallic carbide heterostructure material NiC/Co_2_C via the mechanochemical-magnesium thermal reduction (MC-MTR) method. Specifically, they ground and mixed nickel nitrate (Ni(NO_3_)_2_·6H_2_O), cobalt nitrate (Co(NO_3_)_2_·6H_2_O), aloe extract (as a reducing agent and carbon source), and NaHCO_3_ (as a flame retardant), then calcined the mixture at 800 °C in a nitrogen atmosphere to generate NiCoO_4_. Subsequently, NiCoO_4_ was mixed with magnesium powder and NaHCO_3_, reduced in a closed reactor at 600 °C for 2 h, and the by-products (Na_2_CO_3_, MgO) were removed by washing with hydrochloric acid, finally obtaining a porous NiC/Co_2_C heterostructure. In 1 M KOH electrolyte, NiC/Co_2_C exhibits excellent OER performance, with a minimum overpotential of 221 mV@10 mA/cm^2^ and a Tafel slope of 43 mV/dec. The efficient catalytic mechanism of the NiC/Co_2_C heterostructure in the OER stems from its unique dynamic phase transition and electronic synergistic effect. Triggered by a potential ≥1.54 V, in situ electrochemical reconstruction occurs on the material surface: cobalt atoms in Co_2_C are rapidly oxidized to the γ-CoOOH active phase, and nickel ions in NiC migrate to the surface of γ-CoOOH to form a nickel-enriched layer. This reconstruction process significantly enhances the OH adsorption capacity by introducing oxygen vacancies and activating lattice oxygen to participate in the reaction by stabilizing high-valent cobalt species (with a Co^3+^/Co^2+^ ratio as high as 6.50), laying the foundation for the LOM [46]. In addition, electron redistribution at the heterointerfaces is the core of improving intrinsic activity. XPS analysis shows that nickel atoms inject electrons into cobalt sites through bridging oxygen (O^2−^) (the binding energy of Ni 2p increases while that of Co 2p decreases), which lowers the d-band center of Co^3+^ and optimizes the adsorption energy of oxygen intermediates (*O, OOH). This “Ni→ O→ Co” charge transfer channel makes the cobalt sites in a moderately electron-enriched state, which not only accelerates the deprotonation of OOH but also promotes O-O coupling [47].

Munawar et al. [48] constructed a heterostructure material (Co_2_C-NiTe/SS) where cobalt carbide (Co_2_C) nanosheets cover nickel telluride (NiTe) nanosheets on a conductive stainless steel substrate (SS) via the hydrothermal synthesis method. This material exhibits excellent bifunctional catalytic activity in 1 M KOH. For the HER, it requires an overpotential of only 227 mV at a current density of 10 mA/cm^2^ with a Tafel slope of 48 mV/dec. For the OER, the overpotential is 279 mV at 10 mA/cm^2^ with a Tafel slope of 53 mV/dec. The mechanism behind the efficient bifunctional catalytic activity of Co_2_C-NiTe for both HER and OER in alkaline media lies in its heterointerfacial engineering and dynamic electronic synergistic effects. The vertically oriented NiTe nanosheets constructed on the conductive stainless steel substrate via the hydrothermal method provide a three-dimensional supporting framework for the Co_2_C nano-network. This nanosheet interpenetrating structure creates abundant interfacial active sites, significantly enhancing the adsorption capacity of reactants. XPS analysis confirms the presence of Co^3+^/Co^2+^ (780.8/795.2 eV) and Ni^3+^/Ni^2+^ (855.0/873.1 eV) redox couples at the interface. Among them, cobalt species dominate the OER process, while nickel species optimize the HER pathway, and the bimetallic synergy reduces the water splitting energy barrier. In addition, the electron redistribution induced by the cobalt carbide coating is the core for accelerating reaction kinetics. TEM shows that Co_2_C nanosheets are anchored on the NiTe surface with a coherent interface, forming a metal carbide electron channel. This structure promotes the carbon atoms in Co_2_C to gain electrons from the tellurium atoms in NiTe (the binding energy of the C-Co bond in XPS decreases to 284.3 eV), and at the same time, the d-band center of nickel shifts upward, which collectively optimizes the adsorption energy of intermediates. For OER, the deprotonation energy barrier of OOH at cobalt sites is reduced. Meanwhile, the oxygen vacancy defects on the material surface and interfacial strain synergistically enhance the intrinsic activity. Electrochemical impedance spectroscopy (EIS) reveals that the charge transfer resistance of Co_2_C-NiTe is only 1.8 Ω, which stems from two mechanisms. Firstly, the high conductivity of carbides accelerates electron migration; secondly, the local built-in electric field formed at the interface drives the rapid diffusion of OH^−^/H^+^. In situ Raman spectroscopy further confirms that under a potential of 1.5 V, γ-NiOOH and CoOOH* active phases are preferentially formed at the interface, and their reconstruction process achieves a self-sustaining catalytic cycle through the coupling of Co^2+^→Co^3+^ oxidation and Ni^3+^→Ni^2+^ reduction [48].

Meng et al. [49] synthesized a composite catalyst where the heterostructure of Co_3_C and CoFe is encapsulated in bamboo-like nitrogen-doped carbon nanotubes (CoFe-Co_3_C@NCNTs) via chemical vapor deposition, using cobalt-iron bimetallic Prussian blue analog (CoFe-PBA) and dicyandiamide (DCDA) as precursors. The specific synthesis route is as follows: Firstly, CoFe-PBA spheres are prepared by co-precipitation, which are then subjected to high-temperature carbonization to form CoFe alloy/carbon composites (CoFe-CSP). Subsequently, the composites are mixed with DCDA for secondary carbonization. At high temperatures, the nitrogen-containing carbon sources generated by the decomposition of DCDA grow into bamboo-like NCNTs under the catalysis of CoFe nanoparticles. Meanwhile, part of the CoFe alloy reacts with carbon to form Co_3_C, ultimately resulting in a unique structure where spherical CoFe-Co_3_C heterojunctions are encapsulated at the ends of NCNTs. In 0.1 M KOH electrolyte, the sample CoFe-Co_3_C@NCNTs-20 exhibits excellent OER catalytic performance, requiring only an overpotential of 320 mV to reach a current density of 10 mA/cm^−2^, with a Tafel slope of 82.3 mV/dec. The interface formed between Co_3_C and CoFe alloy induces electron transfer from CoFe to Co_3_C. In situ X-ray absorption spectroscopy confirms an increase in the Co^3+^/Fe ratio, which optimizes the adsorption energy of OOH intermediates. Additionally, the bamboo-like NCNTs isolate the electrolyte from corrosion; XPS shows that the metal dissolution rate after cycling is less than 2%, and pyridinic nitrogen (398.6 eV) and graphitic nitrogen (401.3 eV) provide additional active sites for ORR [49].

## 4. Nickel Carbides for Electrocatalytic OER

Nickel-based materials have emerged as ideal catalysts for the OER in alkaline electrolytes due to their remarkable cost-effectiveness, excellent electrocatalytic activity, and outstanding environmental adaptability. Their advantages include high nickel element reserves, low price, stable physicochemical properties, and efficient catalytic capacity for OER in alkaline environments. Fu et al. [50] prepared a nickel/carbon composite electrode modified with a polyphenol film (Ni/C-ppl-30-0.1-0.1). This material features a unique, loose, mud-like porous structure. SEM characterization reveals that there are incompletely covered substrate areas and abundant pores on its surface, which are conducive to electrolyte penetration and bubble release. In a 0.1 M H_2_SO_4_ acidic electrolyte, the electrode exhibits excellent OER performance, requiring only an overpotential of 350 mV to drive a current density of 10 mA/cm^2^ [50]. Lin et al. [51] adopted a borate-assisted “pyrolysis-carbonization” strategy. They first synthesized a nickel-iron Prussian blue analogue (NiFe-PBA) precursor via a hydrothermal method and then constructed a hierarchical sponge-like composite electrocatalyst with a matched interface (NiFe-PBA/Ni_3_C(B)) through high-temperature carbonization under the in situ desorption effect of borate ligands. This material is composed of amorphous NiFe-PBA nanosheets and metallic Ni_3_C nanocrystals. In 1.0 M KOH electrolyte, the catalyst exhibits excellent OER performance, requiring only an overpotential of 196 mV to drive a current density of 10 mA/cm^2^, with a Tafel slope as low as 30.1 mV/dec [51].

To enhance the performance of transition metal-based electrocatalysts, researchers mainly focus on two aspects: increasing the number of active sites and enhancing the intrinsic activity of individual sites [52,53]. In terms of regulating the number of active sites, it can be achieved through morphological engineering strategies, such as designing nanostructures, precisely controlling the geometric shape of materials, and constructing multi-dimensional composite systems to expose more active interfaces. For enhancing the intrinsic activity of sites, it relies on the optimization of electronic structure, such as constructing alloy systems or core–shell heterostructures. By adjusting the position of the d-band center and charge transfer efficiency, the adsorption and desorption kinetics of reaction intermediates are strengthened.

**Figure 4:**
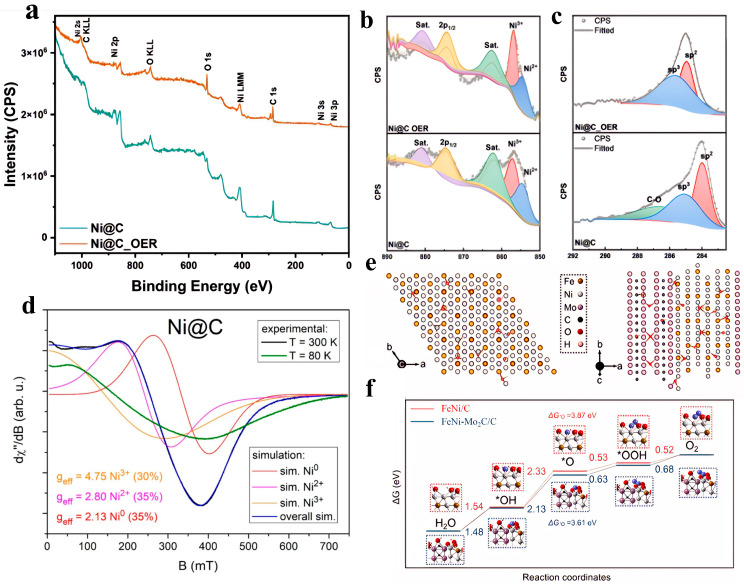
XPS measurements for Ni@C and Ni@C_OER. (**a**) Survey scan, (**b**) Ni 2p core level spectra, and (**c**) C 1s core level spectra. (**d**) EPR spectrum of Ni@C compound. Reproduced with permission [54]. (**e**) Surface-oxidized atomic structure models of FeNi alloys (left) and FeNi–Mo_2_C composite (right). (**f**) The calculated free-energy diagrams of OER processes for FeNi/C and FeNi–Mo_2_C/C. The insets are atomic structure models with oxidized surfaces for OER paths in FeNi/C and FeNi–Mo_2_C/C. Reproduced with permission [55].

Mijowska et al. [54] (Figure 4a–d) synthesized nickel-based carbon composites (Ni@C) through one-step pyrolysis. The structural feature of this material is that nickel nanoparticles are encapsulated by a 2–60 nm thick multi-layer graphite carbon shell and uniformly dispersed on the carbon matrix platform, forming a core–shell structure. In a 1 M KOH alkaline electrolyte, Ni@C exhibits excellent OER performance, with an overpotential of 170.1 mV at 10 mA/cm^2^ and a Tafel slope of 49.0 mV/dec, indicating efficient reaction kinetics. The electrochemically active surface area (ECSA) is as high as 964.7 cm^2^, providing abundant active sites. In addition, the material maintains 81.1% of its activity in a 100-h constant current test, and the overpotential is only 320.8 mV at a high current density of 100 mA/cm^2^, highlighting its industrial-grade stability. It is worth mentioning that the carbon shell plays three performance-optimizing roles: promoting electron transfer, inhibiting the agglomeration of nickel particles, and shielding against electrolyte corrosion. Meanwhile, the in situ formed Ni^3+^ active centers and the carbon carrier synergistically optimize the reaction path [54]. Wang et al. [55] synthesized a carbon layer-supported heterophasic composite catalyst of FeNi alloy and Mo_2_C (FeNi-Mo_2_C/C) via a strategy of high-temperature annealing of precursor mixtures under an argon atmosphere. In a 1 M KOH electrolyte, this material exhibits excellent OER performance, with an overpotential of only 288 mV at a current density of 10 mA/cm^2^ and a Tafel slope of 38.8 mV/dec. In this material, the introduction of the Mo_2_C heterophase effectively inhibits the high-temperature agglomeration of the FeNi alloy, reduces its average particle size, and thus significantly increases the number of electrochemically active sites. Meanwhile, the heterogeneous interface induces significant charge redistribution. XPS and differential charge density analysis confirm that electrons migrate from Fe atoms to Ni atoms and Mo_2_C, resulting in a negative shift in the 2p_3/2_ binding energy of Ni and a positive shift in the 2p_3/2_ binding energy of Fe. This electronic reconstruction optimizes the intrinsic activity of the active sites: on the one hand, it promotes the in situ formation of high-activity surface metal oxides; on the other hand, DFT calculations (Figure 4e,f) confirm that it reduces the reaction free energy of the rate-determining step (*OH→*O) from 2.33 eV to 2.13 eV, significantly lowering the reaction energy barrier [55].

**Figure 5:**
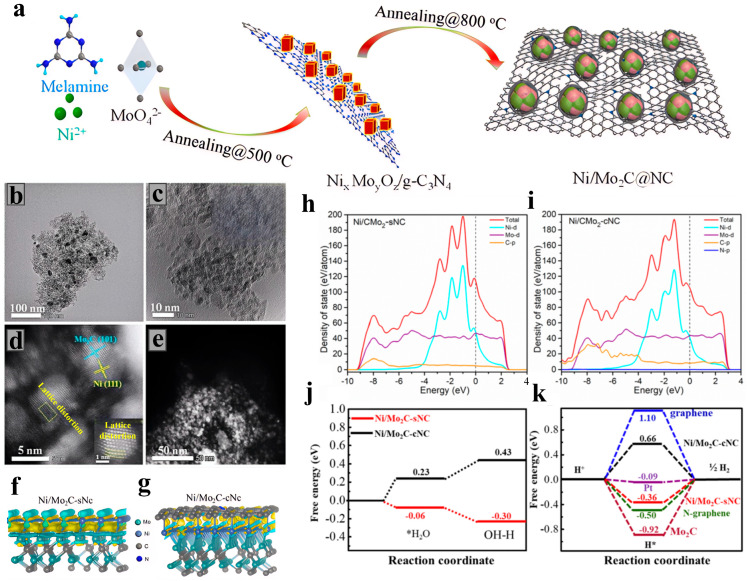
(**a**) Schematic illustration of the synthesis process of the Ni/Mo_2_C@NC hybrid. (**b**) TEM images of the Ni/Mo_2_C@NC-0.15 catalyst. Inset in (**c**): The particle size distribution of the sample. (**d**,**e**) High-angle annular dark-field scanning TEM (HAADF-STEM) image. (**f**,**g**) Side views of charge density differences in Ni/Mo_2_C-sNC and Ni/Mo_2_C-cNC hybrids. (**h**,**i**) Total and partial electronic density of states calculated for Ni (111) and Mo_2_C (001) in Ni/Mo_2_C-sNC and Ni/Mo_2_C-cNC interfaces, respectively. (**j**) Calculated free energy diagrams of water adsorption and dissociation. (**k**) Calculated free energy diagram of H adsorption for graphene, N-doped graphene, Mo_2_C, Ni/Mo_2_C-sNC, Ni/Mo_2_C-cNC, and Pt. Reproduced with permission [56].

Ge et al. [56] successfully prepared an electrocatalyst of multi-interface nickel/molybdenum carbide hybrid nanoparticles anchored on nitrogen-doped carbon nanosheets (Ni/Mo_2_C@NC) using a solid-state co-reduction method. Through a two-step programmed annealing process, they precisely regulated the ratio of nickel-molybdenum precursors, with the optimized ratio being Ni/Mo_2_C@NC-0.15, and constructed a core–shell heterostructure. The nickel/molybdenum carbide nanoparticles (with an average particle size of 4.2 nm) are encapsulated by an N-doped carbon layer, forming abundant Ni/Mo_2_C heterointerfaces (Figure 5a). TEM confirms that the structure has clear lattice fringes and tight core–shell contact (Figure 5b–e). XPS analysis reveals that the electron transfer from Ni to Mo_2_C leads to a negative shift in the binding energy of Ni 2p and a positive shift in that of Mo 3d, confirming the interfacial electronic coupling effect. In 1.0 M KOH electrolyte, this catalyst exhibits excellent bifunctional performance: for the HER, the overpotential is only 91 mV at 10 mA/cm^2^, with a Tafel slope of 74 mV/dec, and it maintains high activity with an overpotential of 131 mV at 10 mA/cm^2^ in an acidic environment (0.5 M H_2_SO_4_); for the OER, the overpotential is 366 mV at 10 mA/cm^2^, and the Tafel slope is 101 mV/dec. Its excellent electrochemical activity stems from the electron reconstruction induced by the heterointerfaces. As shown in Figure 5f–k, DFT calculations confirm that the Ni/Mo_2_C interface optimizes the adsorption energy of water molecules (ΔG_H2O_ = −0.06 eV) and the adsorption free energy of hydrogen intermediates (ΔG_H_ = −0.36 eV) through electron penetration through a single layer of graphene, significantly reducing the water dissociation energy barrier (0.43 eV) and accelerating the kinetics of the Volmer-Heyrovsky reaction [56]. Roy et al. [57] adopted a one-step in situ carbonization strategy. They synthesized the material by ball-milling and mixing phosphomolybdic acid/phosphotungstic acid, nickel chloride, and melamine precursors, followed by pyrolysis at 900 °C in a vacuum-sealed quartz tube. Melamine serves as both the carbon source and the nitrogen source. During the pyrolysis process, a nitrogen-doped graphitic carbon (NGC) support is formed, and nickel atoms are induced to diffuse into the lattices of molybdenum carbide/tungsten carbide, ultimately obtaining nickel-doped molybdenum carbide/tungsten carbide nanoparticles (Ni-MoC/WC@NGC). In 0.5 M KOH, the OER overpotential is 310 mV at 10 mA/cm^2^, with a Tafel slope of 57 mV/dec [57].

## 5. Iron Carbides for Electrocatalytic OER

Iron carbides and carbon composites, with their unique electron-rich metal-carbon bonds and graphitic carbon encapsulation layers, have emerged as ideal carriers for optimizing catalyst matrices. Among them, Fe_3_C nanoparticles act through interfacial electronic coupling and chemical interactions [58,59,60]. The material exhibited excellent OER performance; this enhancement stems from Fe_3_C’s regulation of the d-band center of active sites, optimizing the adsorption energy barrier of oxygen intermediates (*OOH), while the carbon layer suppresses the oxidative deactivation of metal particles. Abbas et al. employed a carbonization synthesis strategy, preparing a core–shell structured catalyst by heat-treating a mixture of adenine and ferric chloride under an argon atmosphere. They synthesized Fe_3_C@C and further doped it with nitrogen to obtain Fe_3_C@C-N. In a 1 M KOH electrolyte, an overpotential of 361 mV is required to reach a current density of 10 mA/cm^2^, with a Tafel slope of 89 mV/dec [61]. Li et al. [62] adopted a thermal reduction carbonization method to prepare a molybdenum-iron-copper carbide hybrid catalyst (Mo_2_C-FeCu) through dopamine-assisted self-assembly and high-temperature treatment. This material has a three-phase heterostructure: the inner core is Mo_2_C nanoparticles, which serve to provide catalytic active sites; the matrix is an FeCu alloy, forming a conductive network to optimize electron transport; and the surface is a graphitic carbon layer, which encapsulates to protect the active components. In 1 M KOH electrolyte, Mo_2_C-FeCu exhibits excellent OER performance, requiring only an overpotential of 238 mV to reach 10 mA/cm^2^, with a Tafel slope of 41.3 mV/dec [62]. Chen et al. [63] successfully synthesized a hollow chain-like nitrogen-doped carbon nanotube/iron carbide composite catalyst (FN-B-800) by high-temperature carbonization of a mixture of ferric chloride and melamine under an argon atmosphere with the assistance of polyvinylpyrrolidone (PVP) regulation. In 0.1 M KOH electrolyte, FN-B-800 exhibits excellent OER electrocatalytic performance, with an overpotential of only 380 mV at a current density of 10 mA/cm^2^ and a Tafel slope of 58.9 mV/dec [63].

**Figure 6:**
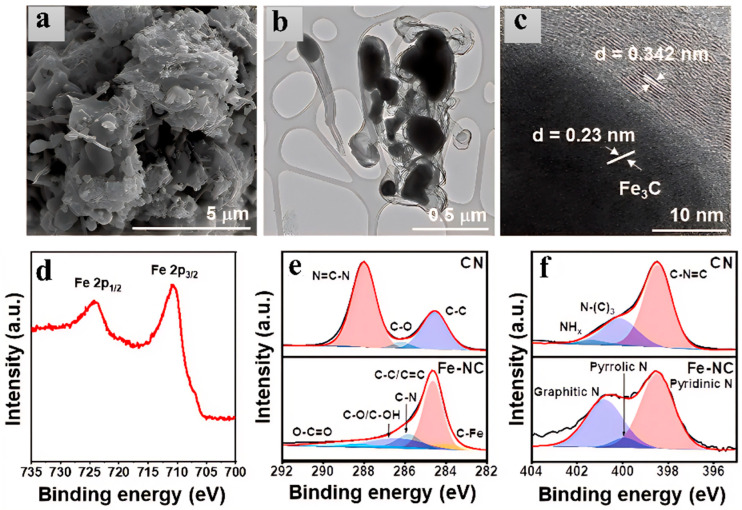
(**a**) An SEM image and (**b**,**c**) TEM images of Fe–NC. (**d**) An XPS Fe 2p spectrum of Fe–NC. Deconvoluted XPS spectra of UCN and Fe–NC: (**e**) C 1s and (**f**) N 1s. Reproduced with permission [69].

Regarding the problems commonly existing in traditional iron carbide-based catalysts, such as poor conductivity, limited specific surface area, high charge transfer resistance, and low density of active sites [64,65]. By introducing graphene as a carrier to construct a three-dimensional porous composite structure, the bottleneck of its catalytic efficiency can be effectively broken through [66]. The highly conductive network of graphene significantly reduces the charge transfer resistance, while its unique wrinkled and layered topological structure greatly increases the specific surface area, providing high-density dispersed anchoring sites for iron carbide nanoparticles, thereby significantly improving the exposure of active sites. This three-dimensional porous composite system inhibits the agglomeration and sintering of iron carbide particles through the geometric confinement effect, optimizes the mass transfer path of the reaction, and synergistically enhances the catalytic activity and stability [67]. For example, Qiao et al. [68] used polyvinylidene fluoride (PVDF), melamine, and iron acetate as precursors to synthesize iron carbide nanoparticles embedded in edge-enriched, nitrogen-fluorine co-doped graphene/carbon nanotube hybrid materials (Fe_3_C@N-F-GCNTs) through high-temperature carbonization at 900 °C under an argon atmosphere. In this material, graphene and bamboo-like carbon nanotubes are covalently bonded to form a three-dimensional conductive network (Figure 6a,b), with a large number of nano-pores on its surface (marked by red circles in Figure 6b) and a high specific surface area of 421 m^2^/g (Figure 6c), providing abundant edge active sites. It exhibits excellent OER electrocatalytic performance in 0.1 M KOH electrolyte, with an overpotential of 432 mV at a current density of 10 mA/cm^2^ and a Tafel slope of 140 mV/dec [68].

Choi et al. [69] used urea and iron (II) chloride tetrahydrate (Fe(II)Cl_2_(H_2_O)_4_) as precursors. After mixing and stirring in ethanol and vacuum drying, they pyrolyzed the mixture at 550 °C for 5 h under a nitrogen atmosphere to synthesize a hybrid material (Fe-NC) in which iron carbide nanoparticles are dispersed on a nitrogen-doped carbon support. As shown in Figure 6d–f, the Fe_3_C particles have a particle size of about several hundred nanometers. Some Fe_3_C particles are encapsulated by a 2–3 nm thick NC layer, while others are directly exposed on the NC surface, providing active sites for the OER. The BET specific surface area is 57 m^2^/g, higher than that of the control group UCN (47 m^2^/g). The mesoporous structure (average pore diameter 27 nm) promotes mass transfer. It exhibits excellent OER performance in 1 M KOH electrolyte, with an overpotential of only 545 mV at a current density of 10 mA/cm^2^ and a Tafel slope as low as 82 mV/dec (Figure 6) [69].

Jaiswal et al. [70] prepared electrocatalysts using a one-step pyrolysis strategy. With ferrocene, toluene, and thiourea as precursors, a series of core–shell nanostructures of sulfur and nitrogen co-doped carbon-encapsulated iron carbide (Fe_3_C@C-SN) were synthesized in a nitrogen atmosphere by regulating the reaction temperature (600–900 °C), thiourea doping amount (25–100 mg), and heating rate (10–20 °C/min). Among them, the Fe_3_C@C-SN/25-800 sample prepared at 800 °C with a thiourea addition of 25 mg exhibited the optimal catalytic performance. The prepared catalyst has a core–shell nanostructure with Fe_3_C nanoparticles as the core and S, N co-doped graphitized carbon as the shell. Fe_3_C, as the main active center, provides catalytic sites. The carbon shell introduces abundant defect sites due to heteroatom doping and improves conductivity. The overall structure shows mesoporous characteristics, with a BET specific surface area of 56 m^2^/g. Electrochemical performance tests showed that Fe_3_C@C-SN/25-800 exhibited excellent OER activity in 0.1 M KOH, with a potential of 1.653 V (vs RHE) required to reach a current density of 10 mA/cm^2^. Its high catalytic activity stems from the synergistic effect of multiple factors. Firstly, during the pyrolysis process, ferrocene decomposes to form the Fe_3_C core, toluene synchronously cracks to form a carbon skeleton, and thiourea realizes the co-doping of S and N elements in the carbon skeleton through pyrolysis. N exists in forms such as pyridinic and pyrrolic types, and S exists in forms such as thiophene type, which together regulate the electronic structure of the carbon shell. In addition, the interface interaction between Fe_3_C and doped carbon forms Fe-N_x_ and N_x_-C active sites, which significantly enhance the adsorption strength of O_2_ and reaction intermediates (such as *OH, *OOH) and accelerate the ORR reaction kinetics. At the same time, the graphitized carbon shell effectively improves the electron transport efficiency. EIS tests show that its charge transfer resistance is 4.33 Ω cm^2^, which promotes interface charge transfer. The mesoporous structure and high specific surface area provide sufficient exposure of active sites and efficient mass transfer channels for the reaction [70].

Chen et al. [71] synthesized the electrocatalyst using a two-step method (liquid-phase impregnation combined with physical activation). Firstly, pretreated Sargassum Horneri (SH) was subjected to liquid-phase impregnation with FeCl_3_ to form the SH@FeCl_3_ precursor. Subsequently, through CO_2_ physical activation and H_2_ treatment at 800 °C, a hierarchically porous composite material of nitrogen-doped biochar supported Fe_7_C_3_ nanoparticles (NHAC@Fe_7_C_3_) was prepared. Among them, the sample with a mass ratio of seaweed to FeCl_3_ of 3:1 (NHAC@Fe_7_C_3_ = 3:1) exhibited the optimal performance. The synthesized material has a composite structure where Fe_7_C_3_ nanoparticles are anchored on nitrogen-doped biochar, featuring a unique wrinkled surface and hierarchical pore structure. The nitrogen-doped biochar provides good conductivity, and Fe_7_C_3_, as the active component, is uniformly dispersed. Overall, it simultaneously possesses a high specific surface area and efficient mass transfer channels. Its electrochemical performance is excellent; in 1 M KOH electrolyte, the OER overpotential of NHAC@Fe_7_C_3_ = 3:1 is only 181 mV at a current density of 10 mA/cm^2^, with a Tafel slope as low as 86 mV/dec. Moreover, it can stably operate for more than 33 h at 10 mA/cm^2^, showing better activity and durability than most non-noble metal catalysts. The high catalytic performance of this material stems from the synergistic effect of multiple factors. Firstly, liquid-phase impregnation allows Fe^3+^ to uniformly penetrate into the seaweed matrix. After high-temperature activation, the formed Fe_7_C_3_ nanoparticles are tightly combined with the nitrogen-doped biochar. DFT calculations confirm that the synergistic effect between the two reduces the free energy of the rate-determining step of OER by regulating the electronic structure, providing sufficient active sites for the reaction. In addition, the hierarchical pore structure not only increases the specific surface area, exposing more active sites, but also accelerates the transport of electrolyte ions and the adsorption of reactants, promoting the mass transfer process. Meanwhile, the good conductivity of the nitrogen-doped biochar provides an efficient channel for electron transport, reduces charge transfer resistance, and further accelerates reaction kinetics [71].

Mayakrishnan et al. [72] developed a high-performance bifunctional electrocatalyst based on the Fe_2_N-Fe_3_C heterostructure, which was prepared using a dual-effect synthesis strategy: with eggs as the natural nitrogen and carbon source and FeCl_3_ as the iron source, Fe_2_N-Fe_3_C heterostructured nanospheres (8HFe_2_N-Fe_3_C) were constructed via liquid-phase impregnation combined with a one-step pyrolysis method (at 800 °C). Among them, the prepared sample exhibited the optimal catalytic performance when the mass ratio of seaweed to FeCl_3_ was 3:1. This material is composed of Fe_2_N-Fe_3_C tightly coupled heterojunction nanospheres anchored on a nitrogen-doped biochar substrate, featuring a hierarchical pore structure dominated by mesopores and a high specific surface area of up to 180 m^2^/g; the elements Fe, N, and C are evenly distributed, with the presence of Fe-N bonds and abundant defects. Furthermore, in 1 M KOH electrolyte, the material demonstrated excellent electrochemical performance: in the HER process, the overpotential required to reach a current density of 10 mA/cm^2^ was 151 mV, with a Tafel slope of 91 mV/dec; in the OER process, the overpotential corresponding to a current density of 10 mA/cm^2^ was 251 mV, with a Tafel slope of 93 mV/dec; in the overall water splitting reaction, the voltage needed to achieve a current density of 10 mA/cm^2^ was only 1.56 V. In addition, the material could operate stably for more than 50 h in HER, OER, and overall water splitting processes, with negligible activity decay. The optimal catalytic activity was achieved when the catalyst loading was optimized to 0.28 mg/cm^2^. Its high catalytic activity stems from the synergistic effect of multiple factors: Firstly, the heterointerfaces between Fe_2_N and Fe_3_C realize charge redistribution through Fe-N bonds, where Fe_2_N acts as an electron transport channel to promote charge transfer, and Fe_3_C provides abundant active sites; the two synergistically reduce the energy barrier for water splitting (with the activation energy as low as 3.65 kJ/mol). Secondly, the hierarchical pore structure and high specific surface area increase the number of exposed active sites, accelerate electrolyte mass transfer, and ensure the efficient progress of the reaction. Thirdly, nitrogen doping and surface defects can regulate the electronic structure, optimize the adsorption energy of reaction intermediates (*OH, *OOH), and reduce the energy barrier of the OER rate-determining step (*O→*OOH), thereby improving the kinetic rate. Surface defect engineering can directionally induce the in situ oxidation process. Taking Fe_2_N-Fe_3_C/nitrogen-doped biochar as an example, the interfacial Fe-N bonds trigger charge redistribution, prompting iron sites to oxidize into an FeOOH active layer under OER potential. In situ Raman spectroscopy confirms that this controlled oxidation is accompanied by the proliferation of oxygen vacancies, which simultaneously enhances the OH adsorption energy and stabilizes high-valent iron species, reducing the OER overpotential to 251 mV@10 mA/cm^2^ [46,72].

Li et al. [73] developed a multifunctional electrocatalyst (VN/Fe_3_C@NCNT) that activates iron carbide catalytic sites through vanadium nitride (VN). This catalyst is prepared via a one-step pyrolysis method, using dicyandiamide as the carbon and nitrogen source, Fe(NO_3_)_3_ and NH_4_VO_3_ as the iron and vanadium sources, respectively, to in situ construct VN/Fe_3_C heterojunctions in nitrogen-doped carbon nanotubes (NCNT) at 800 °C. In 1 M KOH electrolyte, VN/Fe_3_C@NCNT exhibits excellent multifunctional electrochemical performance: in the HER, the overpotential to reach a current density of 10 mA/cm^2^ is 151 mV, with a Tafel slope of 68.2 mV/dec; in the OER, the overpotential corresponding to a current density of 10 mA/cm^2^ is 337 mV, with a Tafel slope of 90 mV/dec; the half-wave potential of the ORR is 0.875 V (vs. RHE). After 600 cycles, the performance decay is less than 5%. The loading amount of the electrode catalytic layer is 0.3 mg/cm^2^. Its high-efficiency catalytic performance stems from the activation of Fe_3_C active sites by VN and the synergistic effect of multiple components, with the specific mechanism as follows: Firstly, VN, as an electron regulation center, reconstructs the active sites of Fe_3_C. DFT calculations show that in HER, VN injects electrons into the CFe sites of Fe_3_C, optimizing the H* adsorption free energy from 0.38 eV to 0.14 eV, making CFe the main active center for HER; in ORR, VN reduces the adsorption energy of Fe sites for O_2_ (from −0.75 eV to −0.92 eV) and accelerates the cleavage of the O-O bond (the energy barrier is reduced by 0.3 eV). In addition, the heterogeneous interface promotes charge redistribution. In XPS analysis, the Fe 2p_3/2_ binding energy shifts positively by 0.5 eV, and the V 2p_3/2_ binding energy shifts negatively by 0.3 eV, confirming the electron transfer from Fe to V, which synchronously enhances the redox activity of Fe_3_C—the d-band center of Fe sites shifts upward by 0.17 eV, strengthening the adsorption of *OOH intermediates, while the electron-deficient surface of VN (V^4+^/V^3+^ = 1.2) can stabilize reaction intermediates. Meanwhile, NCNT, as a conductive network, accelerates mass transfer and electron conduction: the graphitized carbon layer (I_D_/I_G_ = 0.89) reduces the Rct to 4.7 Ω, and the mesoporous structure improves electrolyte permeability, ensuring that the OER overpotential is only 312 mV at a high current density of 50 mA/cm^2^ [73].

**Figure 7:**
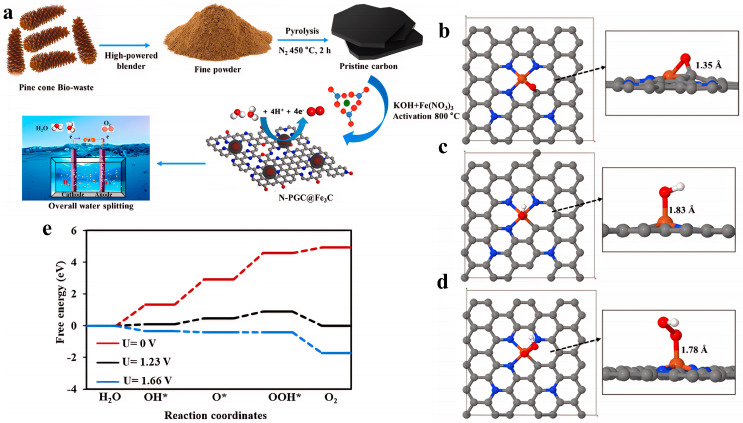
(**a**) Schematic depiction of the synthesis procedure for the N-PGC@Fe_3_C catalysts. Computed structures of (**b**) O*, (**c**) OH*, and (**d**) OOH* on the N-PGC@Fe_3_C-3 catalysts. (**e**) Computed OER free-energy diagram of O*, OH*, and OOH* intermediates at the N-PGC@Fe_3_C-3 catalysts at U = 0, 1.23, and 1.66 V. Reproduced with permission [74].

To address the research issues of insufficient active sites and poor conductivity in Fe-N-C catalysts, the Yoong Ahm Kim team proposed a solution involving a biomass-derived nitrogen-doped porous graphitic carbon-encapsulated Fe_3_C core–shell structure (N-PGC@Fe_3_C). They synthesized a hierarchical mesoporous N-PGC@Fe_3_C-3 catalyst through KOH activation of pine cone biomass carbon precursor, impregnation with Fe(NO_3_)_3_, and high-temperature carbonization (Figure 7a). Its OER electrochemical performance is as follows: in 1 M KOH, only an overpotential of 143.8 mV is required to reach a current density of 20 mA/cm^2^, with a Tafel slope as low as 109.8 mV/dec, and the activity decays after 2000 cycles. The mechanism behind the enhanced performance of N-PGC@Fe_3_C materials in the OER stems from the synergistic effect of their unique core–shell structure, electronic regulation, and hierarchical pores. As shown in Figure 5a, after the biomass-derived carbon is impregnated with iron salt and activated with KOH, high-temperature carbonization forms a core–shell structure (N-PGC@Fe_3_C) where Fe_3_C nanoparticles are wrapped in nitrogen-doped porous graphitic carbon (N-PGC). The interfacial coupling between the Fe_3_C core and the nitrogen-doped carbon shell induces strong electronic interactions. As shown in Figure 7b–e, DFT calculations further reveal that the hybridization of Fe-3d orbitals with N-2p orbitals shifts the d-band center upward, reducing the energy barrier for *OOH formation and lowering the limiting potential of the rate-determining step (*O→*OOH) to 1.66 V. Meanwhile, the hierarchical mesoporous structure and three-dimensional conductive network promote electrolyte penetration and electron transport. Electrochemical impedance spectroscopy shows that the charge transfer resistance of N-PGC@Fe_3_C-3 is significantly reduced, while the high ECSA and abundant edge defects expose more active sites [74].

## 6. Other Metal Carbides

In bimetallic catalytic systems, the combination of iron (Fe) with other transition metals (such as Mo, Co, etc.) significantly enhances electrocatalytic performance through a multi-level synergistic mechanism, and their activity and stability are generally superior to those of single-metal catalysts [75,76,77]. The core reason lies in the electronic structure regulation at the heterogeneous interface and the synergistic effect of multiple active sites. Specifically, the bimetallic system forms mixed crystal phases and structural disorder, exposing a high density of edge sites and enhancing intrinsic activity [78,79].

**Figure 8:**
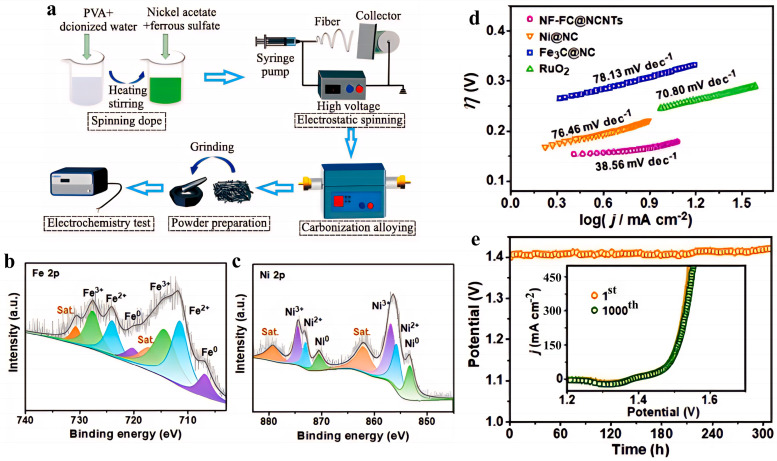
(**a**) Preparation process flow chart of electrospinning-heat-treated samples. High-resolution XPS spectrum of Fe 2p. Reproduced with permission [80]. (**b**) and Ni 2p (**c**) of NF-FC@NCNTs after OER stability test. OER catalytic performances of the catalysts in 1.0 M KOH, (**d**) Tafel plots. (**e**) Chronopotentiometric curve of the NF-FC@NCNTs catalyst at 10 mA/cm^2^ (Inset: LSV plots of the 1st scan and the 1000th cycle). Reproduced with permission [81].

Li et al. [80] adopted a synergistic strategy of electrospinning and atmosphere heat treatment, and synthesized a multiphase composite nanofiber catalyst (Ni_3_Fe/Ni_4_S_3_/Ni/C) by regulating the heat treatment temperature (600 °C/800 °C/1000 °C) (Figure 8a). It exhibits excellent OER performance in 1 M KOH electrolyte. The sample treated at 1000 °C (S-1000) shows the best performance, with an overpotential of only 298 mV at 10 mA/cm^2^ and a Tafel slope as low as 74 mV/dec [80]. Liu et al. [81] employed a one-step pyrolysis and in situ growth strategy to successfully synthesize a self-supported bifunctional electrocatalyst, Ni_3_Fe-Fe_3_C@NCNTs/NFF, using nickel-iron foam (NiFe foam, NFF) as the substrate and DCDA as the carbon and nitrogen source via single-step high-temperature pyrolysis. This material exhibits excellent bifunctional electrochemical performance in an alkaline electrolyte (1 M KOH). For the HER, the overpotentials are 80 mV at 10 mA/cm^2^ and 291 mV at 500 mA/cm^2^, with a Tafel slope of 74 mV/dec. For the OER, the overpotentials are 171 mV at 10 mA/cm^2^ and 315 mV at 500 mA/cm^2^, with a Tafel slope of 38.56 mV/dec (Figure 8) [81].

Zhang et al. [82] synthesized a heterostructured catalyst with abundant interfaces, specifically carbon nanofibers embedded with WC_1−x_/Mo_2_C nanoparticles (denoted as WC_1−x_/Mo_2_C@CNF), using electrospinning combined with heat treatment. In this catalyst, WC_1−x_ and Mo_2_C form tightly coupled heterojunctions and are uniformly dispersed in the carbon nanofibers. The material exhibits hydrophilicity and flexibility, making it suitable for application as a binder-free electrode. In addition, this material exhibits excellent electrochemical performance in a 1 M KOH electrolyte. In the HER, the overpotential required to reach a current density of 10 mA/cm^2^ is 97 mV, with a Tafel slope of 42.9 mV/dec. It can operate stably for 60 h, and the Faraday efficiency is close to 100%. For the OER, the overpotential corresponding to a current density of 10 mA/cm^2^ is 243 mV, the Tafel slope is 79.8 mV/dec, and it can run stably for 40 h. In the overall water splitting reaction, only a voltage of 1.56 V is required to achieve a current density of 10 mA/cm^2^. Meanwhile, its high-efficiency catalytic performance stems from the synergistic effect of multiple factors: Firstly, the work function difference between WC_1-x_ and Mo_2_C (4.78 eV for WC_1−x_ and 4.41 eV for Mo_2_C) induces the formation of a built-in electric field, which drives the transfer of electrons from Mo_2_C to WC_1−x_, resulting in an asymmetric charge distribution at the interface. This electron transfer behavior is confirmed by XPS and XANES characterizations, showing a negative shift in the binding energy of W and a positive shift in the binding energy of Mo. Secondly, this charge redistribution regulates the d-band center, causing the d-band center of Mo to shift to a higher energy level and that of W to shift to a lower energy level. According to the d-band center theory, this change optimizes the adsorption strength of the catalyst for hydrogen/oxygen intermediates (such as *OH, *OOH, and H*), thereby reducing the reaction energy barrier. Specifically, the energy barrier for the rate-determining step of water dissociation in HER is significantly lowered, and the energy barrier for the conversion of intermediates in the rate-determining step of OER is also optimized. In addition, carbon nanofibers as a carrier not only provide a good conductive network, but their hierarchical structure also increases the number of exposed active sites, accelerates the mass transfer of electrolytes, and further improves the catalytic kinetic rate [82].

**Figure 9:**
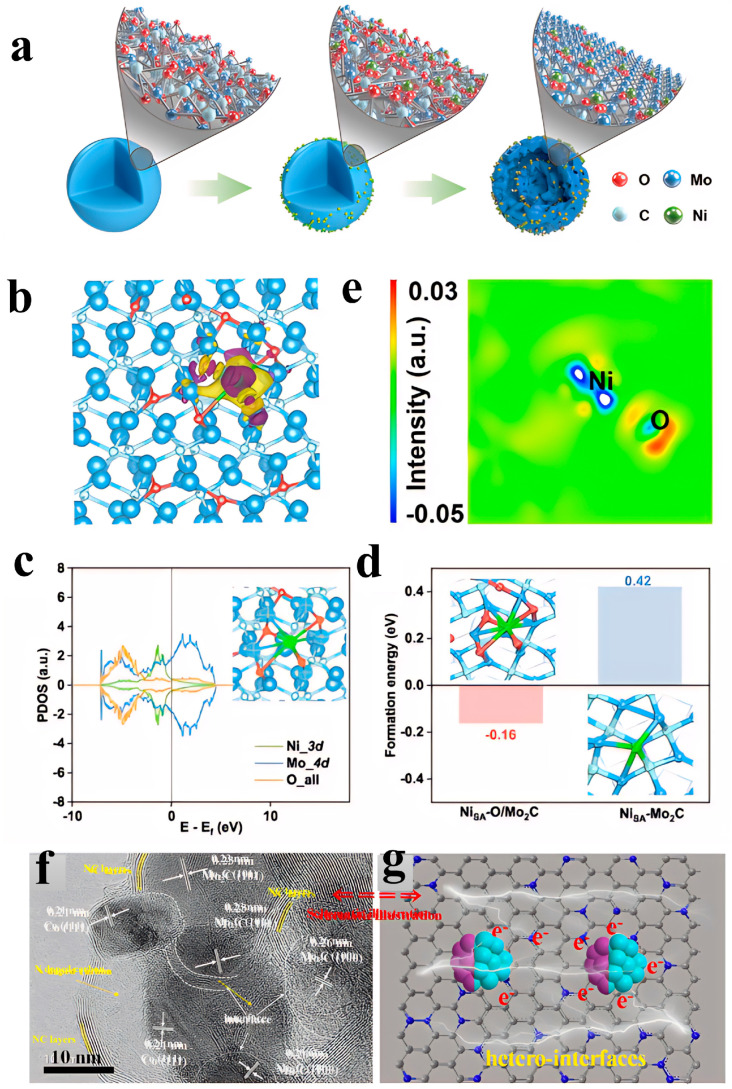
(**a**) Schematic illustration of the fabrication process of the Ni_SA_-O/Mo_2_C electrocatalyst. The two plots of charge density differences of Ni_SA_-O/Mo_2_C: top view of 3D plot (**b**) and 2D display (**c**). The isosurface level was set to 0.003 eÅ^−3^, where charge depletion and accumulation were depicted by purple and yellow, respectively. (**d**) PDOS graph of 3d orbitals of Ni, 4d orbitals of Mo, and all orbitals of O atoms specified by cross marks in Ni_SA_-O/Mo_2_C. (**e**) The chemisorption energies of the Ni atom on the surfaces of Ni_SA_-O/Mo_2_C and Ni_SA_-Mo_2_C. Reproduced with permission [83]. (**f**) HR−TEM images of the Co-Mo_2_C@5NC−4 catalyst. (**g**) Schematic illustration of the structure of Co-Mo_2_C hetero−interfaces. Reproduced with permission [84].

Hou et al. [83] prepared a Mo-based organic nanosphere precursor using the hydrothermal method. After adsorbing Ni ions via an impregnation method, they conducted stepwise heat treatment under air and H_2_/Ar atmospheres (1 h at 300 °C in air, followed by 3 h at 750 °C in a hydrogen-argon atmosphere) to synthesize a bifunctional electrocatalyst (Ni_SA_-O/Mo_2_C), where Ni single atoms are anchored on oxygen-doped Mo_2_C through Ni-O-Mo bridge bonds. This material has a double-shell hollow nanosphere structure with a specific surface area of 64.3 m^2^/g. In addition, the catalyst exhibits excellent electrochemical performance in a 1 M KOH electrolyte: In the HER test, with a loading amount of 0.7 mg/cm^2^ on the glassy carbon electrode, the overpotential required to reach a current density of 10 mA/cm^2^ is 133 mV, the Tafel slope is 83.6 mV/dec, and it can operate stably for 16 h. In the OER test, with a loading of 0.74 mg/cm^2^ on carbon paper, the overpotential corresponding to a current density of 10 mA/cm^2^ is 299 mV, the Tafel slope is 89.36 mV/dec, and it also operates stably for 16 h. In the overall water splitting reaction, when it is used as both the anode and cathode, a voltage of 1.69 V is required to reach a current density of 10 mA/cm^2^. After 120 h, the current density decreases from 10 mA/cm^2^ to 8.5 mA/cm^2^, indicating good stability. Meanwhile, its high-efficiency catalytic performance stems from the synergistic effect of multiple factors: Firstly, Ni forms a strong electronic coupling with oxygen-doped Mo_2_C through Ni-O-Mo bridge bonds, which regulates the electronic structure of Ni. DFT calculations show that the d-band center (−1.66 eV) of Ni_SA_-O/Mo_2_C is lower than that in the case where Ni is directly bonded to Mo_2_C, which weakens the overly strong adsorption of intermediates such as *H and *OH, optimizes the adsorption energy, reduces the energy barrier of the Volmer step in HER (0.55 eV) and the energy barrier of the rate-determining step in OER (*O→*OOH) (1.61 eV), and accelerates the reaction kinetics. In addition, the catalyst undergoes potential-driven reconstruction during the reaction: After HER, Ni remains dispersed as single atoms, with an increase in Ni-O coordination number and a longer Ni-Mo bond length, which is suitable for the adsorption of hydrogen species. After OER, Ni aggregates into small clusters to form Ni-Ni bonds, and the reconstructed structure is more conducive to the conversion of oxygen species. Moreover, partially oxidized Mo_2_C is beneficial to HER, while excessive oxidation promotes OER activity. At the same time, the double-shell hollow structure and high specific surface area increase the number of exposed active sites, accelerate the mass transfer of electrolytes, and further improve the catalytic efficiency (Figure 9a–e) [83].

Huo et al. [84] successfully synthesized a cobalt/molybdenum carbide heterojunction composite catalyst (Co-Mo_2_C@NC) through in situ phase transformation at 800 °C via a one-step pyrolysis-chemical vapor deposition method, using cobalt molybdate (CoMoO_4_) nanorods as the precursor and DCDA as the carbon and nitrogen source. This material has a core–shell structure, where the core consists of a tightly bonded Co/Mo_2_C heterointerface, and the shell is a nitrogen-doped carbon framework. It also features a hierarchical mesoporous structure and a high specific surface area (312 m^2^/g, BET). XRD confirms the coexistence of Mo_2_C and metallic Co. In 1 M KOH electrolyte, the catalyst exhibits excellent electrochemical performance: the HER overpotential is 89 mV@10 mA/cm^2^ with a Tafel slope of 52.7 mV/dec; the OER overpotential is 356 mV@10 mA/cm^2^ with a Tafel slope of 78.3 mV/dec; and the overall water splitting voltage is as low as 1.49 V@10 mA/cm^2^. In terms of the catalytic mechanism, the Co/Mo_2_C heterointerface significantly optimizes reaction kinetics through electron transfer. DFT calculations show that electrons injected from Co into Mo_2_C cause the d-band center of Co to shift upward by 0.25 eV, reducing the H adsorption energy from 0.45 eV to 0.18 eV. Meanwhile, the electron-deficient surface of Mo_2_C (Mo^4+^/Mo^3+^ = 1.5) weakens the Mo-H bond strength, accelerating the Heyrovsky step. In addition, the in situ generated Co LDH/Mo_2_C active interface during the reaction further enhances catalytic efficiency: after the oxidation of the Co surface to CoLDH, the provided OH- adsorption sites on CoLDH synergize with Mo_2_C, reducing the O-O bond breaking energy barrier. At the same time, the dissociation energy of interfacial water molecules decreases by 1.2 eV, and the rate of the Volmer step increases by 3 times. Moreover, the nitrogen-doped carbon framework enhances the hydrophilicity of the material (contact angle 28°) through pyridinic nitrogen (398.6 eV), promoting electrolyte penetration, while the graphitic carbon layer effectively inhibits the agglomeration of Co/Mo_2_C particles (Figure 9f,g) [84].

Kawashima et al. [85] systematically evaluated the performance and dynamic evolution behavior of cubic vanadium carbide (V_8_C_7_) micro particles as an OER electrocatalyst in an alkaline medium (1 M KOH) through a strategy combining experiments and theoretical calculations. For material synthesis, a simple drop-coating method was adopted: commercial V_8_C_7_ powder and carbon black were mixed at a mass ratio of 4:1, ultrasonically dispersed in a solution of isopropanol and Nafion to form an ink, and then loaded onto a glassy carbon electrode with a catalyst loading of 0.49 mg/cm^2^. This catalyst exhibited excellent OER performance in 1.0 M KOH: the overpotential (η) required to reach a current density of 10 mA/cm^2^ was 458 mV, and the Tafel slope was approximately 78 mV/dec. Through in situ SEM-EDX and XRD analyses, this study found that V_8_C_7_ particles undergo anisotropic morphological transformation during the OER process: the initially irregular spherical particles gradually evolve into a cuboid structure. Among the crystal planes, the (110) and (111) planes undergo selective dissolution, while the (100), (010), and (001) planes are stably exposed. DFT calculations showed that the (110) and (111) planes of V_8_C_7_ cannot stably adsorb the key intermediate (HOO*), leading to severe auto-oxidation and dissolution into VO_4_^3−^ ions. In contrast, the (100), (010), and (001) planes can stably adsorb HO*/O* and are partially oxidized to layered vanadium oxyhydroxide (VOOH), thereby forming OER active sites [85].

## 7. Carbides in Acidic Media

Currently, research on TMC-based OER catalysts mainly focuses on alkaline electrolyte systems (e.g., 1 M KOH). However, acidic electrolytes (e.g., H_2_SO_4_, H_3_PO_4_) offer higher energy conversion efficiency in practical devices such as proton exchange membrane water electrolyzers (PEMWEs). Therefore, the poor performance of TMCs in acidic environments has become a key obstacle to their industrialization. This section will systematically sort out the performance limitations of TMCs in acidic OER and analyze the underlying causes from the perspectives of materials chemistry and reaction mechanism.

Transition metal carbides often exhibit poor performance in acidic electrolytes, mainly reflected in low activity, insufficient stability, and slow kinetics [86]. Specifically, their catalytic activity is far inferior to that of noble metal-based catalysts; in OER in particular, a high overpotential is required for activation, resulting in low energy efficiency. Meanwhile, in acidic media, TMCs are vulnerable to carbon corrosion, demetallation, and free radical attack, which lead to rapid degradation of the catalyst structure and irreversible loss of performance.

In the preparation and synthesis of materials, it is of great importance to regulate the poor performance in acidic electrolytes. This can be achieved through the following approaches: constructing heterostructures to optimize electron transfer pathways and enhance the kinetics of H* adsorption/desorption in acidic environments; introducing non-metallic atoms (N, P, S) to adjust the Fermi level of TMCs and alleviate carbon corrosion and metal dissolution in acidic media; using highly conductive carbon matrices as supports to inhibit particle agglomeration, improve charge transfer efficiency, and address structural degradation issues in acidic environments; and developing core–shell coating and gradient carbonization technologies to target carbon corrosion and metal dissolution in acidic media [87].

In the industrial application of acidic electrolytes, carbide-based catalysts are regarded as candidate materials to replace platinum; however, their practical application still faces significant challenges, with insufficient activity and poor structural stability being particularly prominent. More seriously, the acidic environment can trigger the electrochemical dissolution of metal components in carbides. Additionally, the porous structure of carbides is prone to degradation, and carbides are susceptible to carbon corrosion and metal dissolution during long-term operation, leading to the loss of active sites and performance degradation. These drawbacks collectively limit the large-scale application of carbides in proton exchange membrane water electrolyzers, and it is urgent to optimize their electronic structure and corrosion resistance through strategies such as alloying or heteroatom doping [88,89].

## 8. Conclusions and Outlook

This paper reviews the development history of transition metal carbide electrocatalytic OER catalysts and systematically introduces the research progress of TMCs in the field of electrocatalytic oxygen evolution reaction in recent years (Table 1). To improve the electrocatalytic OER performance, researchers have developed a variety of regulation strategies, including morphology control, heterostructure construction, component regulation, and conductive substrate modification. However, the field still faces key challenges in terms of stability, mechanistic clarity, and large-scale synthesis. These issues urgently need to be addressed and are also important directions for future research.

The stability problem remains a core bottleneck restricting its practical application. Although TMCs show good stability in alkaline environments, they are prone to oxidative dissolution and carbon matrix corrosion under acidic or high-potential conditions, which seriously limits their long-term service performance. Strategies such as carbon encapsulation and heterointerface engineering can effectively alleviate material degradation by protecting active sites and promoting charge redistribution. However, under industrial-level current densities, the structural instability of materials will be further aggravated, so it is urgent to conduct in-depth research on the interaction mechanism between materials and electrolytes. Future research should prioritize the use of in situ techniques to monitor the surface reconstruction process and develop structures that can resist agglomeration and oxidative leaching under different pH conditions.

The deepening of mechanistic understanding is a key bridge connecting theory and application. Existing studies have shown that the OER reaction on transition metal carbides follows the AEM or the LOM. The interfacial charge transfer effect (such as the directional migration of electrons from Co to Mo_2_C) plays a key role in optimizing the adsorption energy barrier of intermediates. DFT analysis confirms that heterojunctions can change the position of the d-band center and reduce the energy barrier of the O→OOH step. However, the dynamic surface evolution of carbides to oxides or hydroxides during the OER process greatly increases the difficulty of identifying active sites. It is urgent to use advanced in situ characterization techniques (such as Raman spectroscopy and X-ray absorption spectroscopy) to analyze these transient processes, especially to clarify the participation mechanism of lattice oxygen and quantify the influence of defects or vacancies on reaction energetics.

The development of synthesis technology needs to focus on improving the ability to regulate precision. The currently used methods, such as pyrolysis, hydrothermal growth, and metal-organic framework (MOF)-derived carbonization, can prepare high-activity nanostructures (such as hydrangea-like Co/Mo_2_C@NC), but they have limitations such as irregular morphology, ligand toxicity (e.g., release of PH_3_), and poor regulation of heteroatom doping. Atomically precise design, such as single-atom catalysts (e.g., Mn SA-CoP NNs) or vacancy engineering regulation, provides an effective way to maximize the exposure rate of active sites and mass transfer efficiency.

## Figures and Tables

**Table 1:** Comparative table of OER performance of transition metal carbides.

Catalyst	Catalyst Dose	Electrolyte	OER Overpotential	Tafel Slope	Ref.
Co-Co_2_C/CC	——	1.0 M KOH	261 mV@10 mA·cm^−2^	61 mV·dec^−1^	[38]
Fe_x_Co_1−x_Cy	0.5 mg·cm^−2^	1.0 M KOH	420 mV@10 mA·cm^−2^	65 mV·dec^−1^	[40]
CoFe@Co_2_C@Co/NF	——	1.0 M KOH	246 mV@10 mA·cm^−2^	100 mV·dec^−1^	[39]
H-2D Co/Mo_2_C@NC	——	1.0 M KOH	256 mV@10 mA·cm^−2^	48 mV·dec^−1^	[41]
Co-CoO/Ti_3_C_2_-MXene	2 mg·cm^−2^	1.0 M KOH	271 mV @10 mA·cm^−2^	47 mV·dec^−1^	[44]
Co_3_Mo_3_C	2 mg·cm^−2^	5.0 M KOH	1500 mV @ 400·mA·cm^−2^	50.6 mV·dec^−1^	[42]
Co/MoC@NC	2 mg·cm^−2^	1.0 M KOH	279 mV@10 mA·cm^−2^	74 mV·dec^−1^	[43]
Ni/C-ppl-30-0.1-0.1	——	0.1 M H_2_SO_4_	350 mV@10 mA·cm^−2^	74 mV·dec^−1^	[50]
NiFe-PBA/Ni_3_C(B)	1 mg·cm^−2^	1.0 M KOH	196 mV@10 mA·cm^−2^	30.1 mV·dec^−1^	[51]
Ni@C	——	1.0 M KOH	170.1 mV@10 mA·cm^−2^	49.0 mV·dec^−1^	[54]
FeNi-Mo_2_C/C	0.5 mg·cm^−2^	1.0 M KOH	288 mV@10 mA·cm^−2^	42 mV·dec^−1^	[55]
Ni/Mo_2_C@NC	0.2 mg·cm^−2^	1.0 M KOH	366 mV@10 mA·cm^−2^	82 mV·dec^−1^	[56]
Ni-MoC@NGC	0.28 mg·cm^−2^	1.0 M KOH	300 mV@10 mA·cm^−2^	76 mV·dec^−1^	[57]
Fe_3_C@C-N	0.4 mg·cm^−2^	1.0 M KOH	320 mV@10 mA·cm^−2^	68 mV·dec^−1^	[61]
Mo_2_C-FeCu	0.3 mg·cm^−2^	0.1 M Na_2_SO_4_ + 0.05 M NaOH	238 mV@10 mA·cm^−2^	79 mV·dec^−1^	[62]
FN-B-800	0.4 mg·cm^−2^	1.0 M KOH	380 mV@10 mA·cm^−2^	79 mV·dec^−1^	[63]
Fe_3_C@N-F-GCNTs	——	0.1 M KOH	432 mV@10 mA·cm^−2^	140 mV·dec^−1^	[68]
Fe-NC	——	1.0 M KOH	545 mV@10 mA·cm^−2^	82 mV·dec^−1^	[69]
N-PGC@Fe_3_C	——	1.0 M KOH	143.8 mV@20 mA·cm^−2^	58.2 mV·dec^−1^	[74]
Ni_3_Fe/Ni_4_S_3_/Ni/C	——	1.0 M KOH	298 mV@10 mA·cm^−2^	74 mV·dec^−1^	[80]
Ni_3_Fe-Fe_3_C@NCNTs	——	1.0 M KOH	171 mV@10 mA·cm^−2^	38.5 mV·dec^−1^	[81]
CoNiC/CoNibc/SSM	——	1.0 M KOH	253.4 mV@10 mA·cm^−2^	36.0 mV dec^−1^	[45]
NiC/Co_2_C	0.24 mg·cm^−2^	1.0 M KOH	221mV@10 mA·cm^−2^	43 mV·dec^−1^	[47]
Co_2_C-NiTe/SS	——	1.0 M KOH	279 mV@10 mA·cm^−2^	53 mV·dec^−1^	[48]
CoFe-Co_3_C@NCNTs	0.5 mg·cm^−2^	0.1 M KOH	320 mV@10 mA·cm^−2^	82.3 mV·dec^−1^	[49]
NHAC@Fe_7_C^3^	——	1.0 M KOH	181 mV@10 mA·cm^−2^	86 mV·dec^−1^	[71]
VN/Fe_3_C@NCNT	0.3 mg·cm^−2^	1.0M KOH	337 mV@10 mA·cm^−2^	90 mV·dec^−1^	[73]
8HFe_2_N-Fe_3_C	0.28 mg·cm^−2^	1.0M KOH	251 mV@10 mA·cm^−2^	93 mV·dec^−1^	[72]
WC_1−x_/Mo_2_C@CNF	——	1.0M KOH	243 mV@10 mA·cm^−2^	79.8 mV·dec^−1^	[82]
Ni_SA_-O/Mo_2_C	0.74 mg·cm^−2^	1.0 M KOH	299 mV@10 mA·cm^−2^	89.36 mV·dec^−1^	[83]
Co-Mo_2_C@NC	0.5 mg·cm^−2^	1.0M KOH	356 mV@10 mA·cm^−2^	78.3 mV·dec^−1^	[84]
V_8_C_7_	0.49 mg·cm^−2^	1.0M KOH	458 mV@10 mA·cm^−2^	78 mV·dec^−1^	[85]

## Data Availability

No new data were created or analyzed in this study.
